# Rescue of a vaccine strain of peste des petits ruminants virus: *In vivo* evaluation and comparison with standard vaccine

**DOI:** 10.1016/j.vaccine.2014.10.050

**Published:** 2015-01-09

**Authors:** Murali Muniraju, Mana Mahapatra, Hubert Buczkowski, Carrie Batten, Ashley C. Banyard, Satya Parida

**Affiliations:** a The Pirbright Institute, Ash Road, Pirbright, Woking, Surrey GU24 0NF, UK; b Animal and Plant Health Agency, Weybridge, Surrey KT15 3NB, UK

**Keywords:** Rescue, Reverse genetics, PPRV Nigeria 75/1, DIVA vaccine, Complete genome, *In vivo* evaluation

## Abstract

Across the developing world peste des petits ruminants virus places a huge disease burden on agriculture, primarily affecting the production of small ruminant. The disease is most effectively controlled by vaccinating sheep and goats with live attenuated vaccines that provide lifelong immunity. However, the current vaccines and serological tests are unable to enable Differentiation between naturally Infected and Vaccinated Animals (DIVA). This factor precludes meaningful assessment of vaccine coverage and epidemiological surveillance based on serology, in turn reducing the efficiency of control programmes. The availability of a recombinant PPRV vaccine with a proven functionality is a prerequisite for the development of novel vaccines that may enable the development of DIVA tools for PPRV diagnostics. In this study, we have established an efficient reverse genetics system for PPRV Nigeria 75/1 vaccine strain and, further rescued a version of PPRV Nigeria 75/1 vaccine strain that expresses eGFP as a novel transcription cassette and a version of PPRV Nigeria 75/1 vaccine strain with mutations in the haemagglutinin (H) gene to enable DIVA through disruption of binding to H by the C77 monoclonal antibody used in the competitive (c) H-ELISA. All three rescued viruses showed similar growth characteristics *in vitro* in comparison to parent vaccine strain and, following *in vivo* assessment the H mutant provided full protection in goats. Although the C77 monoclonal antibody used in the cH-ELISA was unable to bind to the mutated form of H *in vitro*, the mutation was not sufficient to enable DIVA *in vivo.*

## Highlights

• Rescue of a vaccine strain of peste des petits ruminants virus.
• *In vivo* evaluation of rescued vaccine strain and comparison with standard vaccine.
• 1SStrategy for Differentiating Infected from Vaccinated Animals (DIVA).

## 1 Introduction

Peste des petits ruminants (PPR) is an important infectious viral disease of domestic and wild small ruminants that threatens the food security and sustainable livelihood of farmers across Africa, the Middle East and Asia [1,2]. PPR is emerging in new regions of the world and is causing great economic losses [3]. The causative agent, peste des petits ruminants virus (PPRV) belongs to the family *paramyxoviridae*, genus *morbillivirus* [4] alongside other important viral pathogens such as rinderpest virus (RPV), measles virus (MeV) and canine distemper virus (CDV). The morbillivirus genome is around 16 kbp, and encodes a nucleoprotein (N), phosphoprotein (P), matrix protein (M), fusion protein (F), haemagglutinin protein (H) and a large polymerase (L) protein. The N, F and H proteins are the most immunogenic and following infection virus neutralising antibodies are generated against the H and F glycoproteins whilst a poorly neutralising response is seen to the N protein. The live attenuated vaccine strain PPRV Nigeria 75/1 has been used successfully in the field for decades [5]. The commercially available diagnostic ELISAs are targeted against the N and H proteins and detect antibodies in vaccinated as well as naturally infected animals. No tools currently exist that allow serological Differentiation between Infected and Vaccinated Animals (DIVA). To this end, marker vaccines are a potential solution to the DIVA concept that may play an important role in the reduction of PPRV in endemic regions.

Existing DIVA strategies for PPRV have focussed on developing protein subunit vaccines expressing the PPRV F and or H gene in viral vectors like pox viruses (vaccinia, capripox and fowl pox) and adenoviruses (canine or human type) [6–12]. Here the absence of the PPRV N protein in the vaccine preparation facilitates positive serological identification of infected animals. However, such subunit vaccines often require multiple doses and may have reduced efficacy due to: potential pre-existing immunity to the viral vector; reduced antibody induction through an inability to replicate; the potential for short lived antibody responses; potentially high costs of recombinant vaccine production. An alternative strategy is to use the existing live attenuated PPRV vaccine and manipulate a specific region or epitope of a viral protein to obtain positively and or negatively marked vaccines. Reverse genetics provides a means to manipulate RNA virus genomes through DNA copies (cDNA) of the RNA genome. In this study, a reverse genetics system for PPRV Nigeria 75/1 vaccine strain was established successfully with recovery of virus from cDNA. Further, we have generated a positively marked vaccine by generating a recombinant virus expressing eGFP as a novel transcription cassette. Furthermore we have attempted to negatively mark the vaccine by creating a recombinant that is epitope deleted for part of the anti PPRV H C77 monoclonal antibody binding site, a key component of the current diagnostic competitive H ELISA (c-HELISA). The negatively marked rescued virus was assessed in goats to investigate protection when compared to existing vaccine preparations and to assess DIVA potential.

## 2 Materials and methods

### 2.1 Cells culture

VeroDogSLAMtag (VDS) cells [13] were used for virus rescue and propagation. Cells were cultured in Dulbecco's modified Eagle's medium supplemented with 5% (v/v) foetal calf serum (FCS, Gibco) and penicillin (100 U/ml, Sigma) at 37 °C/5% CO_2_.

All viruses were rescued and grown in VDS cells until the detection of cytopathic effect (CPE) before being freeze-thawed, clarified and stored at −70 °C. The Nigeria 75/1 vaccine was obtained commercially (The Pirbright Institute, UK). Final virus titre was determined by 50% tissue culture infectious dose (TCID_50_) as described previously [14].

### 2.2 Phage display peptide library screening for C77 mAb binding epitope

Anti-PPRV-H C77 monoclonal antibody (mAb) derived from a hybridoma line was purified from cell culture supernatant using the Melon Gel Monoclonal IgG Purification Kit (Pierce). Purified C77 was dialysed against 0.1 M NaHCO_3_ (pH 8.6) and its concentration measured using a BCA Protein Assay Kit (Pierce) [15]. The Ph.D.-12™ Phage Display Peptide Library Kit (New England BioLabs) was used to screen for C77 binding epitopes as described previously [15]. Data obtained from several rounds of panning were analysed using the Vector NTi software package (Invitrogen) and amino acid sequences were mapped to the native PPRV Nigeria75/1 H protein.

### 2.3 Construction of recombinant PPRV cDNA clones

**Fig. 1:**
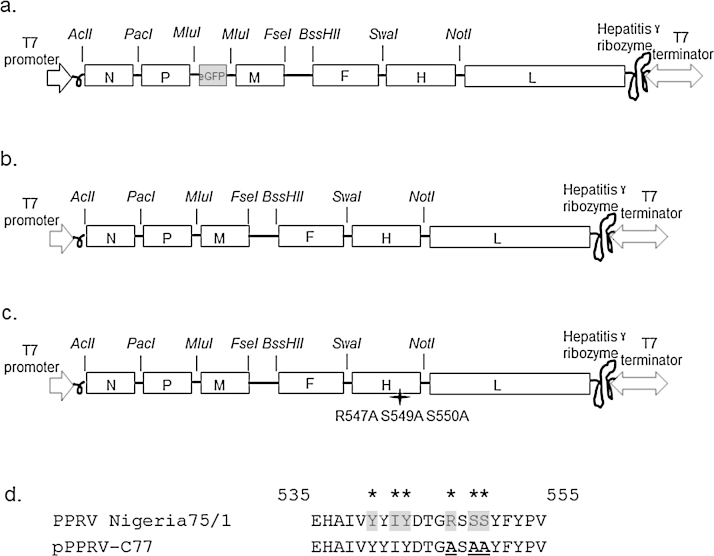
Schematic representation of the PPRV recombinant plasmids and predicted epitopes for C77 monoclonal antibody binding. (a) The synthetic plasmid, pPPRV+GFP, incorporating eGFP as a reporter gene between the P and M genes and restriction enzyme sequences. (b) Plasmid, pPPRV without the eGFP gene incorporating restriction enzyme sequences. (c) Plasmid, pPPRV-C77 incorporating mutations at R547 S549 S550 amino acid positions on the H gene. (d) The proposed epitope (upper strand) for C77 monoclonal antibody binding as determined by phage display, and the mutated H (lower strand). Residues critical to C77 binding are highlighted in grey and indicated by star symbol. Amino acid residues mutated to alanine in plasmid pPPRV-C77 are boldfaced and underlined in lower strand.

The full-length PPRV cDNA plasmids generated were based on the PPRV Nigeria 75/1 vaccine strain (GenBank accession number X74443.2). The plasmid containing the complete PPRV antigenome sequence (15948 nt) with the insertion of eGFP gene was designed and synthesised commercially (DNA2.0, USA) (Fig. 1a). The eGFP reporter gene (822 nt) was introduced to enable rapid evaluation of rescue events, as a separate transcriptional unit between the P and M gene with the authentic 5′ untranslated regions (UTRs) of the M gene and the 3′ UTR of the P gene. Novel restriction enzyme sites were inserted by nucleotide substitution into the untranslated regions (UTRs) of each gene and as such did not affect the total genome length or viral protein sequences. The full length clone was under the expression control of the T7 RNA polymerase promoter and cleaved at the antigenome promoter by the hepatitis delta ribozyme (Fig. 1a). The synthesised plasmid, pPPRV + GFP was sequenced in its entirety to ensure the sequence was 100% identical to the wild type vaccine strain. After the successful recovery of a recombinant PPRV expressing the eGFP gene, the eGFP gene in the plasmid pPPRV + GFP was removed using *Mlu*I to obtain pPPRV (Fig. 1b). To generate a negatively marked vaccine, the C77 binding site mapped through phage display peptide library screening (R547 S549 S550) was altered in the plasmid pPPRV by site directed mutagenesis (R547A S549A S550A) to obtain plasmid pPPRV-C77 (Fig. 1c and d). The epitope mapped by phase display peptide library screening is depicted in Fig. 1d. The helper plasmids required for rescue of recombinant viruses, PPRV Nigeria 75/1 N (pN), P (pP) and L (pL), were cloned under the control of the T7 RNA polymerase promoter in the pGEM3z vector (Promega). All three full length plasmids and helper plasmids were sequenced to ensure that no additional mutations had been incorporated through PCR and cloning.

### 2.4 Transfection and recovery of recombinant PPRV from cDNA

VDS cells (70% confluent) were grown in 6-well plates and infected with T7-polymerase expressing recombinant fowl pox virus at a multiplicity of infection (MOI) of 0.2 as described previously [16]. Cells were washed and transfected with 1 μg of full-length PPRV cDNA plasmid and 1 μg pN, 1 μg pP and 0.05 μg pL using TransFast™ Transfection Reagent (Promega) at a ratio of 6:1 (wt/wt) in a total volume of 0.75 ml of OPTI-MEM I reduced serum medium/well (Gibco). Media was changed on cells at 24 h post transfection and observed for CPE for three days. Rescued viruses were harvested by freeze thawing and further passaged in VDS cells.

### 2.5 *In vitro* characterisation of recombinant viruses

To confirm the identity of rescued viruses, RT-PCR was performed using PPRV specific primers. Total RNA was extracted from rescued viruses at the third passage and analysed by RT-PCR. Primers were designed flanking the eGFP gene and the C77 binding site and amplicons derived from each virus were sequenced to determine the correct sequence were present. Immunoflorescence for the expression of N, H and or eGFP by the rescued viruses was carried out by labelling the N protein with an anti-PPRV-N C11 monoclonal antibody and the H protein with anti-PPRV-H C77 monoclonal antibody (BDSL, UK) and GFP autofluorescence [14,17].

The *in vitro* growth kinetics of the recombinant PPRVs and the parental virus was assessed in a multiple-step growth cycle as described earlier [14,17]. To determine the stability of the inserted eGFP transcriptional unit or the mutated C77 mAb binding site in the recombinant virus genome, the viruses were serial passed in VDS cells for up to nine passages and assessed for the expression of GFP and stable mutation of C77 mAb binding site using RT-PCR followed by sequencing and confocal microscopy.

### 2.6 *In vivo* vaccination and challenge experiment

Animal experiments were conducted according to UK Home Office regulations (Project licence number: 70/6907) and following ethical approval at The Pirbright Institute. European mixed breeds of 12 male goats, aged 6–9 months were randomly split into three groups (*n* = 4/group). Animals in group one (G1, G2, G3 and G4) were immunised with 10^4^ TCID_50_ rPPRV-C77 whilst animals in group two (G7, G8, G9 and G10) received the PPRV Nigeria 75/1 conventional vaccine virus (10^4^ TCID_50_) *via* the sub-cutaneous route. Groups one and two were housed in separate rooms along with two unvaccinated control animals per room (G5, G6, G11 and G12). Animals were monitored daily for 28 days post vaccination. At four weeks post vaccination, the control goats were segregated into a separate room and the animals from all the three treatment groups (*n* = 12) were challenged with a pathogenic PPRV Ivory Coast/89 isolate (10^5^ TCID_50_) by the intranasal route using LMA^®^ MAD Nasal™ Intranasal Mucosal Atomization Device (LMA, San Diego, USA). Animals which developed severe clinical signs were humanely terminated according to an established clinical scorecard [18].

For all animals, rectal temperatures and clinical assessments of animals were conducted twice daily. Ocular swabs were taken in every alternate day post vaccination and challenge to analyse lachrymal secretions of PPRV by real-time RT-PCR using PPRV N gene primers [19]. RNA extraction was achieved using robotic extraction methods (MagNA Pure LC Total Nucleic Acid Isolation Kit, Roche, UK) following the manufacturer's protocols. Heparinised blood samples were collected for virus isolation from peripheral blood mononuclear cells (PBMCs) by co-cultivation with VDS cells [14]. Clotted blood samples were collected for the evaluation of the serum antibody response specific to the PPRV H protein using a PPR Antibody ELISA kit (BDSL, UK) and the development of PPRV neutralising antibodies evaluated using a virus neutralisation test (VNT) [20]. Virus neutralisation titres are expressed in log_10_ and titres >3 were considered positive.

## 3 Results

### 3.1 Recovery and characterisation of recombinant PPRVs from cDNA clones

Infectious recombinant viruses of PPRV Nigeria 75/1, with and without the eGFP (rPPRV + GFP and rPPRV, respectively) and rPPRV-C77 were rescued from cDNA. The CPE characteristic of PPRV infection was observed three days post transfection with 100% efficiency. The CPE observed for all the three recombinant viruses appeared to be identical to that produced by the parental PPRV Nigeria 75/1 vaccine strain. Total RNA isolated from the recovered PPRV recombinants (rPPRV + GFP, rPPRV and rPPRV-C77) at passage three were subjected to RT-PCR using PPRV genome specific primers using a −RT as a control for carry over DNA. The expected amplicon sizes were observed on an agarose gel and sequences were 100% identical to each respective plasmid (data not shown).

**Fig. 2:**
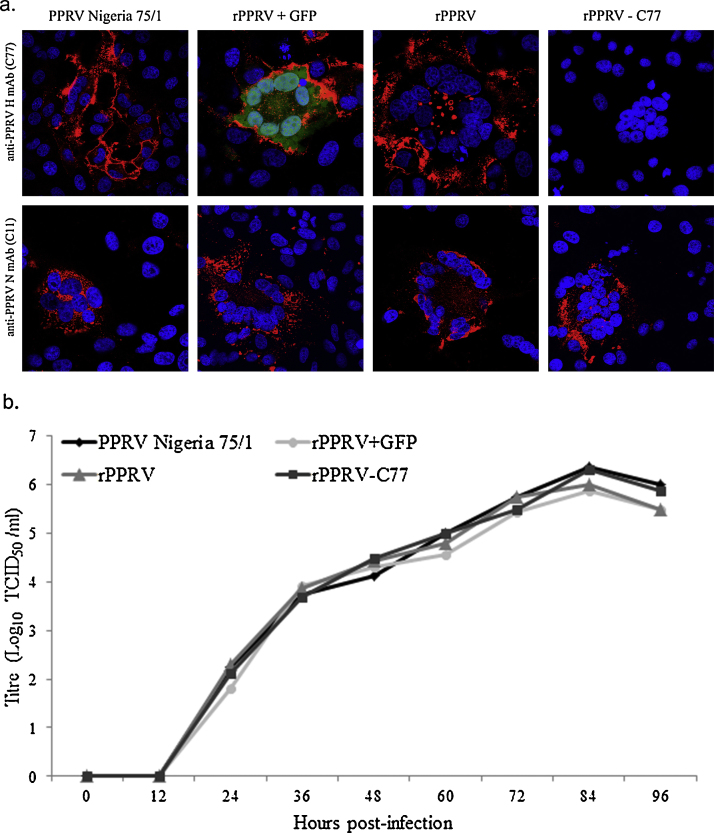
Characterisation of rescued viruses in confocal microscopy and multistep growth study. (a) Expression of PPRV N, and H proteins and/or GFP with C77 mAb binding activity in the PPRV recombinants and parental virus infected cells. VDS cells were infected with viruses at MOI of 0.01 and fixed 24 h post-infection using 4% paraformaldehyde. Cells were stained separately with primary antibodies of mouse anti PPRV H (C77) and mouse anti PPRV N (C11) followed by secondary antibody Alexa Fluor 568 goat anti-mouse (red). Cell nuclei were stained with DAPI (blue) and GFP autoflorescence was visualised (green). The expression pattern of PPRV N and H proteins were comparable between the recombinant and parental virus. Wildtype H protein in PPRV Nigeria 75/1, rPPRV + GFP and rPPRV were detected using the C77 mAb whilst H was not detected using this antibody in cells infected with rPPRV-C77. The autoflorescence of GFP was detected for the rPPRV + GFP virus. (b) Growth kinetics study of recombinant and parental PPRVs in cell culture. Multi-step growth curve was obtained by infecting VDS cells with virus at an MOI 0.01 and grew for different time point and the titre of virus determined (TCID_50_).

Immunofluorescent imaging demonstrated the expression of the PPRV N and H proteins in infected cells following labelling with specific mAbs and expression was comparable to that observed with the commercially available vaccine virus (Fig. 2a). The H protein was detected on the surface of infected cells whilst the N proteins were largely localised in cell cytoplasm. The rPPRV-C77 with the mutated C77 mAb binding site (R547A S549A S550A) was not detected by the anti-PPRV H mAb but virus N protein could be visualised (Fig. 2a). The successful expression of eGFP from an additional transcriptional unit inserted within the PPRV genome was observed through its ability to autofluoresce. Multi-step growth curves were carried out to compare the growth of the recombinant viruses (rPPRV + GFP, rPPRV and rPPRV-C77) with that of the parental vaccine strain (Nigeria 75/1) (Fig. 2b). The recombinant PPRVs grew to a similar titre and rate to that of the parental PPRV Nigeria 75/1 virus.

### 3.2 Clinical protection of goats vaccinated with rPPRV-C77 and conventional PPR vaccine upon challenge with virulent PPRV

**Fig. 3:**
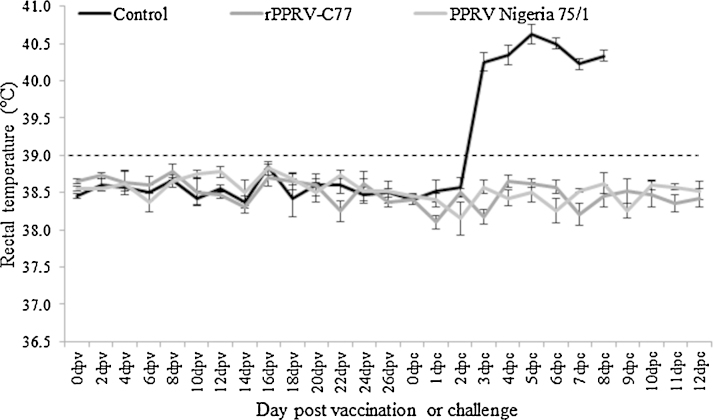
Rectal temperatures of vaccinated (recombinant and parent vaccine virus) and unvaccinated goats upon challenge with virulent PPRV. Temperatures were measured twice per day and are presented as the mean values of the four animals in each group with standard error. (Data provided for every alternative day during vaccination period).

Following vaccination, all animals remained healthy and did not show any vaccination-related adverse effects. Similarly, 28 days post vaccination when the goats were challenged with virulent PPRV, no clinical signs or high rectal temperatures were observed in either the recombinant or conventional vaccinated groups whilst control animals developed severe clinical disease and were humanely terminated at 8 days post challenge (Fig. 3). Control animals housed with groups one and two following vaccination did not seroconvert at 28 days post vaccination. Detection of viral specific RNA and live virus was observed in the eye swabs of control animals during the post challenge period whereas no virus/viral genome was detected from both the vaccinated groups throughout post-challenge period (Tables 1 and 2). The recombinant virus isolated from the PBMCs at 4 and 8 days post vaccination confirmed that the mutated C77 mAb binding site on H protein of rPPRV-C77 had not been altered or reverted back to its native sequence.

**Fig. 4:**
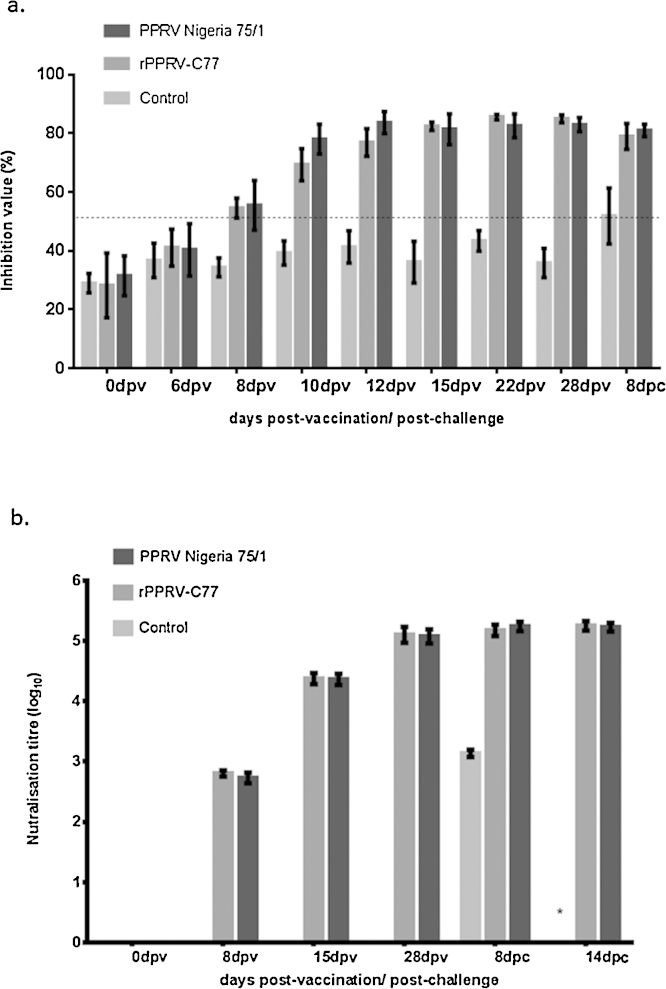
Detection of PPR specific antibodies in vaccinated and challenged goats. (a) Mean percent inhibition values of PPRV H-specific antibody responses in animals vaccinated with the wildtype vaccine, rPPRV-C77 and unvaccinated animals as determined by c-H ELISA. Serum was collected at days 0, 6, 8, 10, 12, 15, 22 and 28 days post vaccination (dpv) as well as on day 8 post-challenge (dpc). (b) Mean virus neutralising titre (expressed in Log _10_) in vaccinated and challenged goats as assessed by VNT. Serum samples were analysed for 0, 8, 15 and 28 days post-vaccination and 8 and 14 days post-challenge. * Represents for the unavailability of serum samples from unvaccinated animals due to their sacrifice on 8 days post-challenge.

The PPRV H-specific antibody response on serum collected from the vaccinated and challenged animals was assessed using the commercially available PPR antibody ELISA kit (BDSL, UK). All animals were sero-negative for PPRV specific antibodies on the day of vaccination. Both the recombinant and parental vaccinated animals were seen positive for PPRV H-specific antibodies starting from day 8 post-vaccination and reached at peak by two week post-vaccination (Fig. 4a). Sera collected during both the vaccination and challenge period were subjected to VNT using homologous PPRV vaccine strains. On the day of vaccination animals were found to be negative for PPR specific antibodies whilst high titre virus neutralising antibodies were detected by second week of vaccination and reached at peak (log 4.76–5.31) on day 28 post vaccination, the day the animals were challenged, in both groups of vaccinated animals (Fig. 4b and supplementary Table 1). All unvaccinated control animals remained negative during this period. Following challenge, titres of antibodies in both groups one and two remained constant at two weeks post challenge. No statistically significant difference in neutralising antibody titre was seen between groups one and two. The unvaccinated animals had developed PPRV specific neutralising antibodies by the eighth day post-challenge, the day they were humanely terminated.

## 4 Discussion

Despite the availability of reverse genetics techniques for other morbilliviruses, a rescue technique for PPRV was lacking until successful rescue was reported almost simultaneously [21,22]. Initial attempts to rescue a field isolate of PPRV were unsuccessful and following extensive investigation, the highly GC rich region of the genome was believed to be a potential bottle neck for viable virus rescue [23]. To overcome potential sequence errors introduced by the techniques involved in stitching together a full length DNA copy of the viral genome artificial DNA synthesis was applied to generate error free plasmids for virus rescue. The insertion of a novel transcription cassette within the genome facilitated ease of virus rescue and potential future evaluation of positive marker genes for DIVA activity. The mutation of residues within H to investigate the potential for a negative marker vaccine demonstrated H gene functionality and stability of the introduced mutations. Likewise, *in vitro* the incorporation of eGFP as a novel transcription cassette and novel restriction enzyme sites in the UTRs of each gene was tolerated. Neither approach affected *in vitro* growth compared to the parental strain. Both approaches had been investigated previously for RPV where *in vitro* development of positively and negatively marked vaccines has been assessed [15,24–26]. Following rescue and passage, CPE observed with both the recombinant viruses were similar to that of the parental virus. The cellular distribution of N and H proteins in recombinant viruses were the same as in cells infected with parental virus and were as expected for PPRV.

Recent studies reporting the rescue of PPRV expressing GFP or FMDV VP1 proteins have been limited to *in vitro* data in their assessment for the protective efficacy against rescued PPR viruses [22,27]. In the present study *in vivo* data has been generated to demonstrate that the recombinant virus is able to generate adequate protection from virulent challenge in the natural host for PPRV, small ruminants. *In vivo* assessment is a necessary prerequisite to determining the utility of recombinant versions of vaccines prior to further development and licencing in the field. Both the parental and mutated (rPPRV-C77) Nigeria 75/1 vaccines provided complete protection against a lethal dose of PPRV challenge. None of the animals in either vaccinated group showed any evidence of PPR disease and survived the challenge with pathogenic PPRV. Furthermore, there was no transmission of vaccine virus to in-contact unvaccinated control animals housed with the vaccinated animals during the 4 week period post vaccination demonstrating that both the parent and mutated (rPPRV-C77) vaccines are highly unlikely to transmit between animals and potentially revert to virulence in the field. Indeed, none of the control animals seroconverted and all developed clinical disease following challenge. High titre PPRV specific serological responses were demonstrated 28 days post-vaccination by both VNT and ELISA for all vaccinated animals. Although the rPPRV-C77 vaccine virus was indistinguishable from the parent vaccine strain in its ability to protect animals, DIVA potential was not fulfilled with the mutations applied to residues within H protein using the current H cELISA. Previous studies with other viral vaccines such as RPV [15], Newcastle disease virus [28] and classical swine fever virus [29] vaccines have postulated that epitope deletion may represent an efficient mechanism to generate DIVA vaccines. However, in the current study although epitope mutation prevented binding of C77 mAb *in vitro* it was not sufficient to enable DIVA *in vivo*. As mapped by phase display screening technique, six critical residues were implicated for C77 binding. Mutation of three critical residues from wild type to alanines (R547, S549 and S550) prevented the binding of mAb to the virus in immunofluorescence studies. These mutations were seen to be stable after several passages of rescued virus in cell culture. Therefore, to keep the changes minimal to the parent virus, we had not mutated other three critical residues (Y540, I542 and Y543) and gone forward for the animal experiment assuming for similar results as *in vitro*. It is likely that these three un-mutated critical residues are additionally involved for the binding and we are looking into this possibility to further explore mutation of the C77 binding region by altering these residues. Another approach is currently underway to further develop a PPRV DIVA test using synthetic peptides specific to the C77 epitope region. If successful to develop a DIVA test, certainly, the provision of a recombinant DIVA PPRV vaccine would be a great boon in the less developed regions of the world where PPRV represents a major obstacle to the development and maintenance of subsistence farming.

## Figures and Tables

**Table 1:** Ct-values obtained from reverse-transcription real-time PCR analysis using the eye swab samples collected from vaccinated and unvaccinated challenged goats.

Treatment group	Goat number	Ct value							
		0dpc	2dpc	4dpc	6dpc	8dpc	10dpc	12dpc	14dpc
rPPRV-C77	G1	–	–	–	–	–	–	–	–
	G2	–	–	–	–	–	–	–	–
	G3	–	–	–	–	–	–	–	–
	G4	–	–	–	–	–	–	–	–
PPRV Nig75/1	G7	–	–	–	–	–	–	–	–
	G8	–	–	–	–	–	–	–	–
	G9	–	–	–	–	–	–	–	–
	G10	–	–	–	–	–	–	–	–
Control	G5	–	–	37.54	29.22	24.21	NA	NA	NA
	G6	–	–	35.78	26.87	25.15	NA	NA	NA
	G11	–	–	38.44	30.45	25.46	NA	NA	NA
	G12	–	–	38.64	28.5	27.62	NA	NA	NA

No Ct-value in real-time PCR is represented as ‘–’; dpc, days post-challenge; NA, not applicable. Ct values more than 40 are considered as negative.

**Table 2:** Virus isolation from eye swab samples of vaccinated and unvaccinated challenged goats.

Treatment group	Goat number	Virus isolation							
		0dpc	2dpc	4dpc	6dpc	8dpc	10dpc	12dpc	14dpc
rPPRV-C77	G1	N	N	N	N	N	N	N	N
	G2	N	N	N	N	N	N	N	N
	G3	N	N	N	N	N	N	N	N
	G4	N	N	N	N	N	N	N	N
PPRV Nig75/1	G7	N	N	N	N	N	N	N	N
	G8	N	N	N	N	N	N	N	N
	G9	N	N	N	N	N	N	N	N
	G10	N	N	N	N	N	N	N	N
Control	G5	N	N	Y	Y	Y	NA	NA	NA
	G6	N	N	Y	Y	Y	NA	NA	NA
	G11	N	N	Y	Y	Y	NA	NA	NA
	G12	N	N	Y	Y	Y	NA	NA	NA

‘N’ represents no virus isolation, ‘Y’ represents virus isolation and ‘NA’ represents not applicable.
