# Supplementary material for: Rescue of a vaccine strain of peste des petits ruminants virus: In vivo evaluation and comparison with standard vaccine
Source: Vaccine. 2015 Jan 9;33(3):465–71. doi: 10.1016/j.vaccine.2014.10.050 (PMC4315076; doi:10.1016/j.vaccine.2014.10.050)
Supplement: Supplementary file 1 [file mmc1.docx]

Supplementary Table1. PPRV specific neutralising antibody titres (expressed in Log_10_) detected in serum of individual vaccinated and unvaccinated challenged goats.

| Treatment group | Goat number | Neutralisation titre (log_10_) | | | | | |
| --- | --- | --- | --- | --- | --- | --- | --- |
|  |  |  |  |  |  |  |  |
|  |  | 0dpv | 8dpv | 15dpv | 28dpv | 8dpc | 14dpc |
| rPPRV-C77 | G1 | 0 | 2.8 | 4.16 | 5.31 | 5.31 | 5.31 |
|  | G2 | 0 | 2.83 | 4.36 | 4.76 | 4.36 | 5.31 |
|  | G3 | 0 | 2.91 | 5.61 | 5.01 | 5.01 | 5.01 |
|  | G4 | 0 | 2.66 | 4.36 | 5.31 | 5.01 | 5.36 |
| PPRV Nig75/1 | G7 | 0 | 2.83 | 4.16 | 5.06 | 5.27 | 5.27 |
|  | G8 | 0 | 2.66 | 4.16 | 5.31 | 5.01 | 5.31 |
|  | G9 | 0 | 2.91 | 4.36 | 4.76 | 5.36 | 5.01 |
|  | G10 | 0 | 2.51 | 4.31 | 5.16 | 5.31 | 5.31 |
| Control | G5 | 0 | 0 | 0 | 0 | 3.31 | NA |
|  | G6 | 0 | 0 | 0 | 0 | 3.06 | NA |
|  | G11 | 0 | 0 | 0 | 0 | 3.11 | NA |
|  | G12 | 0 | 0 | 0 | 0 | 3.06 | NA |
